# Cytoplasmic levels of cFLIP determine a broad susceptibility of breast cancer stem/progenitor-like cells to TRAIL

**DOI:** 10.1186/s12943-015-0478-y

**Published:** 2015-12-15

**Authors:** Rhiannon French, Olivia Hayward, Samuel Jones, William Yang, Richard Clarkson

**Affiliations:** European Cancer Stem Cell Research Institute Cardiff University School of Biosciences, Haydn Ellis Building, Maindy Road, Cardiff, CF24 4HQ UK

**Keywords:** Breast cancer, Cancer stem cells, TRAIL, cFLIP

## Abstract

**Background:**

The clinical application of TRAIL receptor agonists as a novel cancer therapy has been tempered by heterogeneity in tumour responses. This is illustrated in breast cancer, where TRAIL is cytotoxic in cell lines of mesenchymal origin but refractory in lines with an epithelial-like phenotype. However, it is now evident that intra-tumour heterogeneity includes a minority subpopulation of tumour-initiating stem/progenitor-like cells (CSCs) that possess mesenchymal characteristics. We hypothesised therefore that TRAIL may target these phenotypically distinct CSC-like cells that are common to most - if not all - breast cancers, thus impacting on the source of malignancy in a much broader range of breast tumour subtypes than previously envisaged.

**Methods:**

We used colony formation, tumoursphere, flow cytometry and xenograft tumour initiation assays to observe the TRAIL sensitivity of CSC-like cells in a panel of two mesenchymal-like (TRAIL-sensitive) and four epithelial-like (TRAIL-resistant) breast cancer cell lines. Subcellular levels of the endogenous TRAIL inhibitor, cFLIP, were determined by western blot and immunofluorescence microscopy. The effect of the subcellular redistribution of cFLIP on TRAIL sensitivity and Wnt signalling was determined using cFLIP localisation mutants and the TOPFlash reporter assay respectively.

**Results:**

TRAIL universally suppressed the clonal expansion of stem/progenitors in all six of the breast cancer cell lines tested, irrespective of their phenotype or overall sensitivity to TRAIL. A concomitant reduction in tumour initiation was confirmed in the TRAIL-resistant epithelial cell line, MCF-7, following serial dilution xenotransplantation. Furthermore TRAIL sensitivity of breast CSCs was inversely proportional to the relative cytoplasmic levels of cFLIP while overexpression of cFLIP in the cytosol using subcellular localization mutants of cFLIP protected these cells from cytotoxicity. The accumulation of nuclear cFLIP on the other hand did not influence TRAIL cytotoxicity but instead promoted Wnt-dependent signalling.

**Conclusion:**

These data propose a novel role for TRAIL as a selective CSC agent with a broad specificity for both epithelial and mesenchymal breast tumour subtypes. Furthermore we identify a dual role for cFLIP in the maintenance of breast CSC viability, dependent upon its subcellular distribution.

**Electronic supplementary material:**

The online version of this article (doi:10.1186/s12943-015-0478-y) contains supplementary material, which is available to authorized users.

## Background

Breast tumours have intrinsic heterogeneity. Breast cancer cells with stem-like properties make up only a small fraction of a tumour, but owing to their role as instigators of tumourigenesis, are believed to impart the majority of the malignant phenotype [1]. The clinical implication of this cellular heterogeneity is that the efficacy of any therapeutic strategy should be measured by its ability to target stem-like cell sub-populations and thus improve upon long term tumour responses. An additional problem however is that breast cancer stem cells (bCSCs) are resistant to radiotherapy and chemotherapy, and thus by targeting only non-bCSCs, many existing therapeutic regimens may actually increase the proportion of bCSCs within a tumour [2–4]. To date, only a few drugs have been shown to be capable of targeting bCSCs [5, 6]. Therefore there is a clear need to identify effective therapeutics capable of targeting this minority population of highly malignant cells.

Tumour Necrosis Factor Alpha Receptor Apoptosis Inducing Ligand (TRAIL) is a soluble cytokine manufactured by cells of the immune system. TRAIL functions to activate the extrinsic apoptosis pathway in target cells to induce caspase-mediated cell death. This pathway can be inhibited within the cytoplasm by the endogenous Cellular FLICE-Like Inhibitory Protein (cFLIP) that acts by competing with caspase 8 for recruitment to the death inducing signalling complex, formed in response to TRAIL- mediated trimerization of its cognate receptors at the cell surface [7]. Initial reports of the ability of TRAIL to target and induce apoptosis preferentially in cancerous cells led ultimately to the production of clinically approved TRAIL agonists for a number of cancers including lymphoma and lung cancer [8–10]. However, despite their initial promise in pre-clinical studies, overall efficacy of TRAIL agonists in a variety of cancer types has been limited to a minority of partial or complete responders culminating in a limited clinical uptake [10–18]. Thus a better understanding of how and why tumour cells respond differently to TRAIL is called for in order to realise its potential as a therapeutic.

Pre-clinical studies have shown that mesenchymal-like breast cancer cell lines are sensitive to the cytotoxic effects of TRAIL whereas epithelial-like lines are TRAIL-resistant [19]. The cause of this correlation has not yet been explained. With the discovery that at least a proportion of bCSCs commonly exhibit mesenchymal characteristics [20–22], we hypothesised that TRAIL may be able to target the bCSC-like population in a broad range of tumour subtypes. We have shown previously that the stem-like cells of four breast cancer cell lines can be sensitised to TRAIL by inhibition of cFLIP, resulting in decreased tumourigenicity in vivo [23]. This implied that cFLIP may have a key role in determining the sensitivity of bCSCs to TRAIL-induced cytotoxicity. However, due to its homology to the pro-apoptotic caspase 8, it has not yet been possible to develop a selective small molecule inhibitor of cFLIP. Any targeted combination therapy is therefore a long way from the clinic. In contrast, given that TRAIL is available now; the ability to identify those patients most likely to respond favourably to TRAIL as a single agent could lead to its more rapid clinical use. To this end we set out to investigate the susceptibility of bCSCs to TRAIL and the underlying mechanisms which determine TRAIL-susceptibility in breast cancer cell lines.

Here we demonstrate that TRAIL alone was able to inhibit the clonal expansion of all six breast cancer cell lines tested, even those regarded previously as refractory to TRAIL cytotoxicity. The sensitivity of stem/progenitor-like cells (here referred to as bCSCs) to TRAIL was a direct consequence of the sub-cellular re-distribution of cFLIP from the cytoplasm, with an inverse correlation between the level of cytosolic cFLIP and TRAIL susceptibility. Consequently, nuclear cFLIP was not able to protect from TRAIL but instead promoted the Wnt signalling pathway, providing a potential explanation for the previously unexplained association between TRAIL-sensitivity and the mesenchymal phenotype.

These data suggest that the effect of TRAIL may not be limited to mesenchymal subtypes of breast cancer and indicates a potentially beneficial role for TRAIL as a selective CSC agent.

## Methods

All experiments were performed with the approval of the University of Cardiff, School of Biosciences Ethics Committee and animal work was performed in accordance with the Home Office Animals (Scientific Procedures) Act 1986 under project licence 30/2849.

### Constructs

The pcDNA3.1cFLIPL overexpression vector, containing the full length coding sequence of the long form of human c-FLIP (accession number NM_003879) was a kind gift from Dr. Naito (Institute of Molecular and Cellular Biosciences, Tokyo University, Japan). The Mission cFLIPSh vector was a kind gift from Dr. Ladislav Andera (Institute of Molecular Genetics, Prague, Czech Republic). The FOPFlash and TOPFlash reporter plasmids were kind gifts from Dr. Ken Ewan, Cardiff University School of Biosciences.

### Site directed mutagenesis

Site directed mutagenesis was performed on the pcDNA3.1cFLIPL construct, using the QuickChange kit (Stratagene) according to the manufacturer’s instructions to introduce the following mutations; LL439AA and RKR435LIL into the nuclear export and localisation sequences respectively [24]. The following mutagenic primers were used:

- NES: LL439AA
- 3′-CTGTTCTTTCTTTTGCGGGT**CG**G**CG**CCTAGAAGTGTAACTTGAGTTACC-5′
- 5′-GACAAGAAAGAAAACGCCCA**GC**C**GC**GGATCTTCACATTGAACTCAATGG-3′
- NLS: RKR435LIL
- 3′-GGTCTTTGACTCTGTTCTT**GA**TT**A**TG**A**GGGTGAGGACCTAGAAGTGTCG-5′
- 5′-CCAGAAACTGAGACAAGAA**CT**AA**T**AC**T**CCCACTCCTGGATCTTCACAGC-3′

### Cell lines and reagents

The human breast cancer cell lines were obtained from ATCC (MDA-MB-231^ER-HER2-^ SKBR3^HER2+^ BT474 ^ER+HER2+^) and CLS (MDA-MB-468 ^ER-HER2-^ MDA-MB-436 ^ER-HER2-^) except the MCF-7 line which was a kind gift from Dr Julia Gee, Cardiff University. All cell lines were maintained in RPMI 1640 medium (Invitrogen) supplemented with 10 % foetal bovine serum (FBS) (Invitrogen), and 1 % penicillin-streptomycin and L-glutamine mix (Invitrogen). Cells were maintained at 37 °C in 5 % CO_2_.

Recombinant soluble human TRAIL was purchased as super-killer TRAIL from Enzo Life Sciences. Unless otherwise stated , cells were treated with 20 ng/ml TRAIL for 18 h before subsequent assays. The pan-caspase inhibitor Z-Vad-Fmk was purchased from R and D systems and used at a concentration of 20uM. Cells were pre-treated with caspase inhibitor for 1 h prior to treatment with TRAIL. Leptomycin B was purchased from Sigma and used at a concentration of 0.1 ng/ml.

### Overexpression and inhibition of cFLIP in cell lines

Cells were transformed with pcDNA3.1cFLIPL overexpression constructs using lipofectamine 3000 (Invitrogen) according to the manufacturer’s instructions and maintained under antibiotic selection.

In order to inhibit cFLIP, cells were transduced with lentivirus containing shRNA and maintained under antibiotic selection:

- cFLIP shRNA Fwd:
- 5′GATCTCCGGGGATAAATCTGATGTGTCCTCATTACTCGAGTAATGAGGACACATCAGATTTATCCTTTTTA-3′
- cFLIP shRNA Rev:
- 5′AGCTTAAAAAGGATAAATCTGATGTGTCCTCATTACTCGAGTAATGAGGACACATCAGATTTATCCCCGGA-3′

Alternatively, cells were transformed with siRNA using lipofectamine RNAiMax (Invitrogen) according to the manufacturer’s instructions, and cultured in the presence of lipid complexes for 48 h prior to the subsequent assay. The following siRNA oligos were custom designed and purchased from Invitrogen:

- cFLIP 1: 5′-GGAUAAAUCUGAUGUGUCCUCAUUA-3′,
- cFLIP 2: 5′-GAGUGAGGCGAUUUGACCUGCUCAA-3′,
- Scrambled control: 5′-GGACUAAUAGUUGUGCUCCAAUUUA-3′.

### Cell viability assay

Cells to be analysed were cultured in a 96-well plate format. On the day of analysis, 20 μl of Cell Titre Blue reagent (Promega) was added to each well containing 100 μl media. The plate was incubated for 1–4 h at 37 °C 5 % CO_2_, before fluorescence was measured at 560/590 nm using a FLUOstar Optima plate reader (BMG Labtech, Offenberg, Germany).

### Tumoursphere formation assay

Tumoursphere assays were carried out in non-adherent conditions in a serum-free epithelial growth medium (MEBM, Lonza), supplemented with B27 (Invitrogen), 20 ng/ml EGF (Sigma), 5 μg/ml Insulin (Sigma), and 25 μg/ml hydrocortisone (Sigma). Cells were plated in ultra-low attachment plates (Costar, Corning) at a density of 5000 cells/ml. After 7 days tumourspheres were counted, then collected by gentle centrifugation (1100 rpm), dissociated in 0.05 % trypsin, 0.25 % EDTA (Invitrogen) and re-seeded at 5000 cells/ml for subsequent passages [25, 26].

### Colony forming assay

Cells were seeded at a density of 185 cells/well in a 12-well plate format, so that cells were 50 per square cm, and cultured for 10 days [27, 28]. To stain colonies, culture medium was removed and well surface was washed once with PBS. Crystal violet/ethanol mixture was applied to wells and incubated for 15 mins at room temperature. Solution was removed and wells were rinsed twice with PBS. Colonies containing approximately 32 or more cells (having undergone 5 or more divisions) were counted using a GelCount platereader and software (Oxford Optronix) set to count colonies of size 100-1000 μm.

### Tumour initiation in vivo

Serial dilutions of untreated and TRAIL-treated cells were prepared in 50 % Matrigel (BD Biosciences). The cell/Matrigel mix was injected above the lymph nodes of the fourth inguinal mammary fat pad with a Hamilton or insulin needle syringe (BD Micro-Fine). FoxN1Nu/Nu mice were used for all xenograft studies (Harlan Life Sciences, UK). Mice were administered oestrogen in the water during the course of the experiment at a concentration of 10 μg/ml. Mice were examined for tumour growth twice weekly by palpation. Tumours were measured using callipers and tumour volume was measured by the calculation; (tumour width^2^) x tumour length/2. Mice were culled when the entire control group developed tumours at least 5 mm in diameter, and mammary glands, lungs and livers of mice were fixed in formalin for histological analysis.

### Luciferase reporter assay

Following 48 h transfection with TOPFlash, FOPFlash and LacZ reporter plasmids, cells were lysed using 50 μl Passive lysis buffer (Promega) for 30 mins. To each of two white-sided 96-well plates (Costar, Corning), 30 μl of cell lysate was transferred. To assay for LacZ activity, to one plate 30 μl Beta-Glo reagent (Promega) was added and incubated at room temperature with gentle shaking for 20 mins before measuring luminescence output. To the second plate, 30 μl of Bright-Glo reagent (Promega) was added to assay for TOPFlash reporter activity and the luminescence measured immediately using a FLUOstar Optima plate reader (BMG labtech, Offenberg, Germany). TOPFlash reporter activity was normalised to LacZ activity to control for transfection efficiency.

### qRT-PCR analysis

RNA extraction was performed using the Qiagen RNEasy kit according to the manufacturer’s instructions. Resulting RNA was incubated with DNase (Amplicon) for 15 mins before inactivation. RNA concentration was determined using a Nanodrop (GE Healthcare, UK). cDNA was synthesised using MMLV reverse transcriptase (Promega) according to manufacturer’s instructions. Primers were custom designed across exon boundaries using the Primer3 web-based program (http://primer3.ut.ee/) and purchased from Sigma. cFLIP: Forward: 5′-TGATGGCAGAGATTGGTGAG-3′, and reverse 5′-GATTTAGACCAACGGGGTCT-3′. Axin 2: Forward, 5′-AGTGTGAGGTCCACGGAAAC-3′ and reverse 5′-TGGCTGGTGCAAAGACATAG-3′. qPCR was performed using GoTaq polymerase (Promega) and RealTime PCR machine using StepOne software (Applied Biosystems).

### Western blotting

Total cellular or cytoplasmic proteins were extracted from cultured cells and analysed by Western blotting. cFLIP antibodies used were purchased from Santa Cruz (5D8) and R and D systems (AF821). Beta Catenin antibody was purchased from BD Biosciences (610154). To quantitate Western data blots were digitally scanned and the pixel intensity of each band quantified relative to its protein loading control (alpha tubulin or lamin A/C) by densitometry using the program ImageJ (http://imagej.nih.gov/ij/).

### Immunofluorescence

Cells from culture were seeded onto glass coverslips placed in wells of a 48-well culture plate, and allowed to adhere. Cells on coverslips were fixed in 4 % formalin for 15 mins followed by 3 × 5 min washes in PBS, then blocked for 1 h in 10 % normal goat serum (Dako) in PBS with 0.5 % triton-X-100 (Sigma). Cells were then incubated in the primary antibody diluted 1:100, overnight at 4 °C. Following 3 × 5 min washes in PBS, cells were incubated in fluorescence-conjugated secondary antibodies (Alexa Fluors, Invitrogen) diluted 1:400 in 10 % normal goat serum (Dako) and containing DAPI nuclear stain (Invitrogen) for 1 h. Coverslips were then washed 3 × 5 mins in PBS and mounted in Mowiol solution (Sigma). Cells were visualised on a Leica confocal microscope. cFLIP antibody used for fluorescence was purchased from Cell Signalling.

### Flow cytometry

Cells to be analysed were trypsinised, washed and incubated for 1 h in antibody diluted 1:100 in PBS. Flow cytometry was performed on an Accuri Flow Cytometer (BD Biosciences) and analysis of results was performed using a FlowJo software package. APC-conjugated CD44 antibody was purchased from BD Pharmingen, PE-conjugated ALDH1 antibody was purchased from Stratech.

### Statistical analysis

Throughout the article, data are represented as mean and error bars as standard error of a minimum of three independent experiments, unless otherwise stated. Statistical significance was determined using a student’s T-test for two-paired samples. Pearson’s correlation coefficient was used to determine whether data was linearly associated. L-Calc software was used to estimate stem cell number from serial dilutions of tumour xenografts (http://www.stemcell.com/en/Products/All-Products/LCalc-Software.aspx)

## Results

### TRAIL targets breast cancer stem/progenitor-like cells

**Fig. 1:**
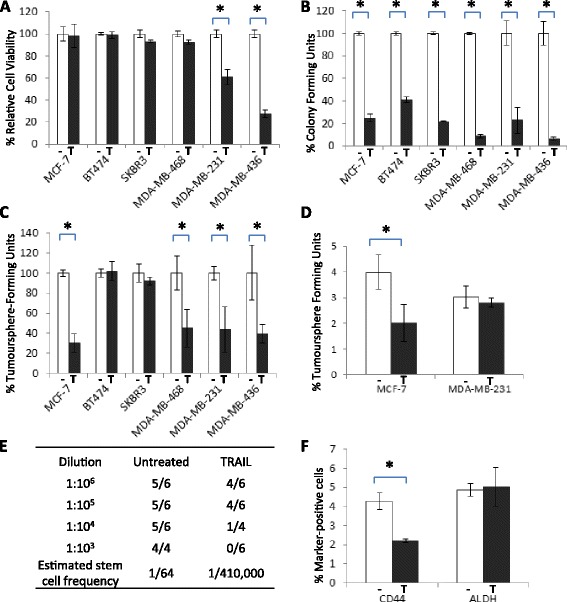
Breast Cancer Stem/progenitors are TRAIL-Sensitive: (**a**) Cell Titre Blue assay performed on 6 cell lines treated for 18 h with 20 ng/ml TRAIL (T) or vehicle control (−). The viability of each line is shown as a percentage of its untreated control. (**b**) Colony-Forming Assay performed on 6 cell lines seeded at low density in the presence or absence of 20 ng/ml TRAIL for 10 days. The proportion of colonies formed is shown as a percentage of its untreated control. (**c**) Tumoursphere Assay performed on each cell line in the presence or absence of 20 ng/ml TRAIL. The proportion of tumourspheres for each cell line is shown as a percentage of its untreated control. (**d**) MCF-7 and MDA-MB-231 cell lines were treated for 18 h in the presence or absence of 20 ng/ml TRAIL, then subjected to tumoursphere Assay performed in the absence of TRAIL. The percentage of tumourspheres is shown relative to the number of cells seeded (**e**) Tumour Initiation in vivo. MCF-7 cells were pre-treated with 20 ng/ml TRAIL for 18 h then implanted into the mammary fat pad of nude mice at serial dilutions. The number of tumours formed relative to transplants was determined by palpation and confirmed by histological analysis at the end of the experiment. An estimation of the proportion of viable stem cells was performed using L-Calc software however a statistically significant comparison was not possible as control data was inconsistent with the model (**f**) Flow Cytometry of MCF-7 cells treated with TRAIL for 18 h and stained with fluorescence-conjugated antibodies to CD44 and ALDH. The percentage of marker-positive cells is shown relative to the number of cells analysed (*p < 0.05, paired t-test, error bars = SEM; all graphs represent means of 3 independent experiments)

Breast cancer cell lines with a mesenchymal-like morphology are relatively more susceptible to the pro-apoptotic effects of TRAIL than their epithelial-like counterparts [19]. On the basis that cancer stem / early progenitor cells (CSCs) have been associated with mesenchymal characteristics we wished to test the hypothesis that TRAIL might preferentially target bCSCs within all breast cancer subtypes, including otherwise resistant epithelial-like cell lines. We used a panel of six breast cancer cell lines that had been reported previously to exhibit different responses to TRAIL-mediated cytotoxicity [19]. We first confirmed that in adherent culture, only those breast cancer cell lines with a mesenchymal-like phenotype (MDA-MB-231 and MDA-MB-436) were sensitive to TRAIL, whereas epithelial-like cell lines (MCF-7, BT474, SKBR3 and MDA-MB-468) were TRAIL-resistant (Fig. 1a).

Colony formation assays were then performed to assess the ability of the minority stem/progenitor cell population within each cell line to expand to form colonies in adherent culture following TRAIL treatment [27, 28]. Each of the six breast cancer cell lines were plated at low density in the presence or absence of TRAIL and colonies allowed to form over a ten day period. Only those colonies containing 32 or more cells (having undergone 5 or more divisions) were counted [28]. TRAIL inhibited colony formation in all six breast cancer cell lines tested (Fig. 1b and Additional file 1: Figure S1A), indicating that the small minority of cells responsible for this clonal expansion were selectively targeted even in a cell line otherwise refractory to TRAIL. Furthermore colony formation could be rescued in the presence of a pan-caspase inhibitor confirming that TRAIL mediated this effect through caspase-dependent cytotoxicity (Additional file 1: Figure S1B).

To determine if TRAIL also suppressed the stem-like traits of self-renewal and anoikis resistance, CSC properties associated with tumour initiation, each of the cell lines were incubated in the presence/absence of TRAIL in non-adherent tumoursphere conditions. Tumoursphere formation was suppressed by TRAIL in four out of six of the breast cancer cell lines tested, including two of the resistant epithelial-like cell lines, MCF-7 and MDA-MB-468 (Fig. 1c and Additional file 1: Figure S1C and D). To confirm that the observed reduction in tumoursphere number was due to a loss of the stem/progenitor compartment, rather than a loss of subsequent clonal expansion, MCF-7 and MDA-MB-231 cells were pre-treated with TRAIL in adherent conditions, prior to seeding of the surviving cells in tumoursphere conditions without TRAIL. MCF-7 tumoursphere- forming cells (TFCs) were significantly more TRAIL-sensitive than the total MCF-7 population whereas TFCs of the TRAIL sensitive MDA-MB-231 line were equally as sensitive to TRAIL as the total cell population (Fig. 1d).

In the two cell lines that exhibited no relative loss in TFC numbers (SKBR3 and BT474: Fig. 1c), continuous TRAIL treatment resulted in a significant reduction in sphere size, confirming our findings in colony forming assays that TRAIL suppressed the clonal expansion of early stem/progenitors (Additional file 1: Figure S1E).

These data combined suggest that although TRAIL may not target stem-like cells directly in all breast tumour lines, the range of tumour types in which bCSCs are susceptible extends to epithelial-like as well as mesenchymal-like cell lines. Furthermore all cell lines tested exhibited attenuated clonal expansion of stem/progenitors together, supporting our hypothesis that TRAIL treatment may target tumour initiation and/or propagation in a broader range of tumour subtypes than assumed previously. To determine the effect of TRAIL on tumour-initiation directly we performed limiting dilution transplantation of one of the most TRAIL-resistant cell lines. MCF-7 cells were treated with TRAIL in vitro for 24 h then viable cells transplanted into the mammary fat pad of mice in the absence of TRAIL. Pre-treatment with TRAIL reduced the number of tumours formed even though equal numbers of viable cells were transplanted compared to untreated controls (Fig. 1e).

The cell surface marker profiles of CD44^+^or ALDH^+^ have been shown previously to enrich for stem/progenitor-like cells with increased capacity for tumour initiation [1, 29]. More recently these marker profiles have been shown to identify two separate subpopulations of stem-like cells [22]. We assessed the ability of TRAIL to target cells with these CSC markers. Treatment of MCF-7 cells with 20 ng/ml TRAIL for 18 h selectively reduced the CD44^+^ population, whereas TRAIL had no significant effect on ALDH^+^ cells (Fig. 1f, and Additional file 1: Figure S1F). As CD44^+^ is thought to identify cells of a more mesenchymal-like nature compared to ALDH [22], our findings are consistent with the established specificity of TRAIL for cells with mesenchymal-like traits [19].

### TRAIL susceptibility correlates with reduced levels of cytoplasmic cFLIP

**Fig. 2:**
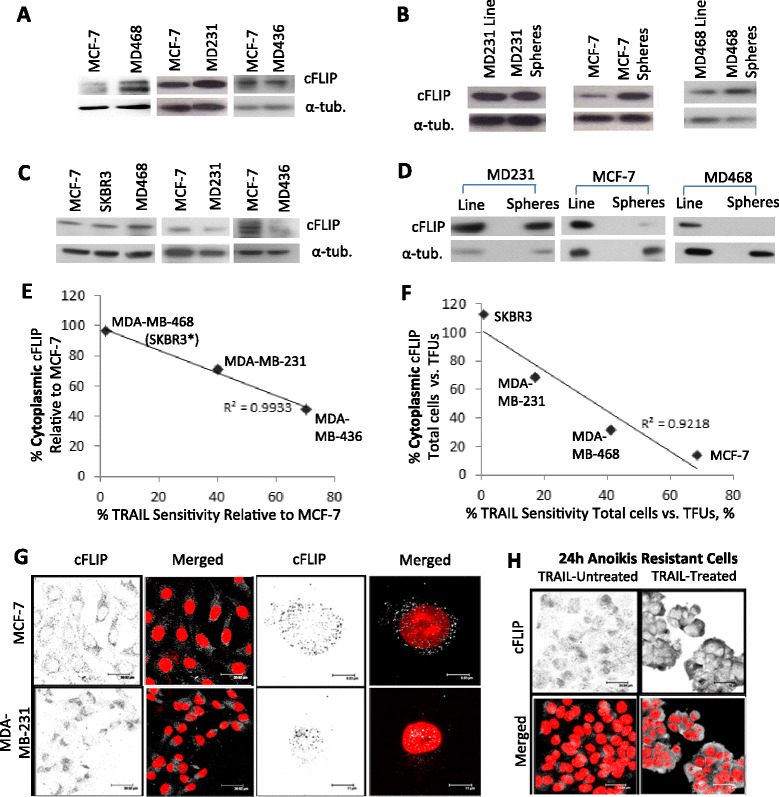
Cytoplasmic cFLIP Levels Correlate with TRAIL Susceptibility (**a**) Representative western blots for cFLIPLong performed on total protein extracts of cell lines MCF-7, MDA-MB-231 (MD231), MDA-MB-468 (MD468) and MDA-MB-436 (MD436). Loading control = α-tubulin. (**b**) Western blots for cFLIPLong performed on total protein extracts of cell lines grown in adherent culture (Line) and non-adherent tumoursphere conditions (Spheres). Loading control = α-tubulin. (**c**) Western blots for cFLIPLong performed on cytoplasmic protein extracts of cell lines. Loading control = α-tubulin. (**d**) Western blots for cFLIPLong performed on cytoplasmic protein extracts of cell lines grown in adherent (Line) or tumoursphere (Spheres) conditions. Loading control = α-tubulin. (**e**) Comparison of relative cytoplasmic cFLIP levels and relative TRAIL-susceptibility (normalised to that of the MCF-7 line). SKBR3 and MDA-MB-468 datapoints overlap (*SKBR3 *n* = 2) (*r* = −0.98 > −0.99, Pearson’s correlation co-efficient). (**f**) Comparison of relative cytoplasmic cFLIP levels and relative TRAIL-susceptibility in bCSC-enriched non-adherent tumourspheres (normalised to that of their bulk cell line) (*r* = −0.96 < −0.95, Pearson correlation co-efficient). (**g**) Immunofluorescence of MCF-7 and MDA-MB-231 cells for cFLIP (cFLIP = grey, DAPI = red) (**h**) Immunofluorescence of anoikis-resistant MCF-7 cells before and after TRAIL-treatment (cFLIP = grey and DAPI = red). All western blots and quantitative data are representative of 3 independent experiments. Quantitation of the mean relative levels of cFLIP in experiments (**a**-**d**) are presented in Additional file 1: Figure S2

While these results indicate a broader TRAIL-specificity of breast cancer stem/progenitors than had been described previously for the total cell populations, the underlying cause of this differential sensitivity to TRAIL is unknown. We have shown previously that inhibition of the “long” isoform of cFLIP sensitises tumoursphere-forming cells to TRAIL [23]. This suggests that the TRAIL-susceptibility of cells with bCSC-like traits is determined, at least in part, by the apoptosis inhibitor cFLIP. Therefore one potential explanation for the observed differences in TRAIL susceptibility between breast cancer cells could be that TRAIL-sensitive cells may contain lower levels of cFLIP. To test this possibility, protein was extracted from breast cancer cell lines grown in adherent culture and from cells suspended in tumoursphere culture for two days to enrich for cells with a stem or progenitor-like phenotype [30]. We observed no direct correlation between the total protein levels of cFLIP and TRAIL-susceptibility in either the adherent (total) populations or the anoikis-resistant (bCSC-enriched) cells, suggesting that total cFLIP levels do not determine TRAIL-susceptibility (Fig. 2a and b and Additional file 1: Figure S2A and B).

In order to inhibit the extrinsic apoptosis pathway, cFLIP must be available in the cytoplasmic compartment of the cell to complex with DISC components and thus interfere with caspase 8 recruitment [7]. Previous studies in lung carcinoma cell lines have found cFLIP (specifically the long form) is able to translocate to the nucleus where it was shown to promote Wnt target gene expression [24]. Given the previously reported association between mesenchymal-like cells and elevated Wnt signalling [31] we inferred that sub-cellular compartmentalisation of cFLIP, rather than total protein levels, might influence TRAIL-sensitivity at the cellular level; more specifically that cytoplasmic cFLIP may be reduced in TRAIL-sensitive cells.

To investigate cytoplasmic levels of cFLIP, nuclear-free cytoplasmic protein fractions were extracted from both the total adherent cell populations and anoikis-resistant populations (bCSC enriched), and levels of cFLIP determined by densitometry of immunoblots. Cytoplasmic cFLIP was reduced in both the MDA-MB-231 and MDA-MB-436 TRAIL-sensitive cell lines but not in TRAIL-resistant MDA-MB-468 cells relative to the MCF-7 cell line (Fig. 2c and Additional file 1: Figure S2C). Cytoplasmic cFLIP was also reduced in TRAIL-sensitive tumourspheres (MCF-7, MDA-MB-231 and MDA-MB-468) but unchanged in TRAIL-resistant tumourspheres of SKBR3 cells (Fig. 2d and Additional file 1: Figure S2D). Thus in both bulk-tumor cells and enriched stem/progenitor populations the relative levels of cytoplasmic cFLIP significantly correlated with TRAIL susceptibility (Fig. 2e and f).

To confirm the relative subcellular distribution of cFLIP, cells were immunostained for cFLIP *in situ* and examined by confocal microscopy in two representative cell lines with differential TRAIL sensitivity. In the TRAIL-sensitive MDA-MB-231 line, cFLIP localised to the nuclear and peri-nuclear compartments, whereas in the TRAIL-resistant MCF-7 line cFLIP staining was punctate and primarily cytoplasmic (Fig. 2g). Analysis of the distribution of staining through the z-plane further confirmed the partial overlap between nuclear content (DAPI) and nuclear/peri-nuclear cFLIP in MDA-MB-231 cells, in contrast to the exclusive distribution of cFLIP and DAPI in MCF-7 cells (Additional file 1: Figure S2E). The anoikis-resistant subpopulation of MCF-7 (tumoursphere) cells, previously demonstrated to be sensitive to TRAIL (Fig. 1c), were also analysed by immunofluorescence. In contrast to the total cell population which exhibited cytoplasmic cFLIP (Fig. 2g), anoikis-resistant cells exhibited nuclear staining and thus a relative decrease in cytoplasmic cFLIP (Fig. 2h, TRAIL-untreated). As expected, treatment with TRAIL reduced tumoursphere number by approximately fifty percent as shown previously (Fig. 1c). The remaining TRAIL-resistant treated (and therefore resistant) cells exhibited a marked elevation in cytoplasmic cFLIP (Fig. 2h, TRAIL-treated). Analysis of the distribution of staining through the z-plane also revealed an overlap between DAPI and cFLIP in anoikis-resistant MCF-7 cells whereas little overlap was apparent in the remaining TRAIL-treated (and therefore TRAIL-resistant) MCF-7 anoikis-resistant cells (Additional file 1: Figure S2F).

Taken together, these data are consistent with the hypothesis that cytoplasmic cFLIP is reduced in TRAIL-sensitive cells.

### Cytoplasmic cFLIP protects cancer stem/progenitors from TRAIL induced cytotoxicity

**Fig. 3:**
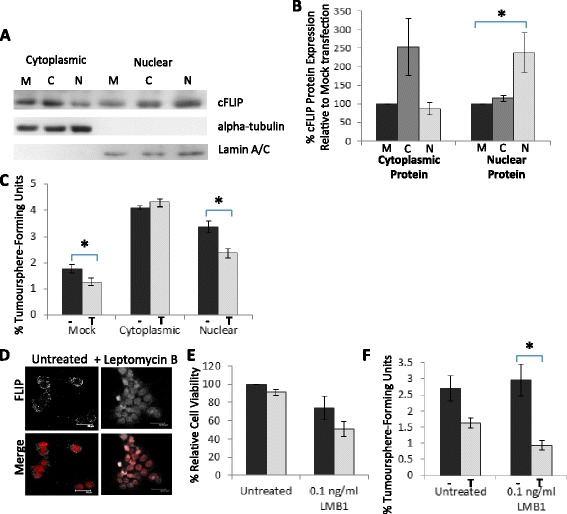
Cytoplasmic but not nuclear cFLIP protects against TRAIL-mediated cell death (**a**) Western blots for cFLIP performed on cytoplasmic and nuclear protein extracts of MCF-7 s transfected with overexpression constructs; mock (*M,* empty vector control), cytoplasmic-localised cFLIP (*C*) and nuclear-localised cFLIP (*N*) (loading controls = α-tubulin and lamin A/C) (**b**) Densitometry analysis of Western blots for cFLIP performed on cytoplasmic and nuclear protein extracts of MCF-7 s expressing mutant cFLIP. Data is average of 3 independent protein samples normalised to Mock control. (**c**) Tumoursphere Assay of MCF-7 cells stably transfected with either mock, cytoplasmic-localised cFLIP or nuclear-localised cFLIP in the presence (T) or absence (−) of 20 ng/ml TRAIL. The percentage of tumourspheres is shown relative to the number of cells seeded (**d**) Immunofluorescence of MCF-7 cells cultured in the presence or absence of 0.1 ng/ml leptomycin-B (LMB1) for 24 h (cFLIP = grey, DAPI = red) (**e**) Cell Titre Blue Assay of MCF-7 cells cultured for 24 h in the presence or absence of 0.1 ng/ml LMB1 before 18 h treatment with 20 ng/ml TRAIL. Cell viability is shown as relative to the untreated control. (**f**) Tumoursphere Assay in the presence (T) or absence (−) of 20 ng/ml TRAIL, of MCF-7 cells cultured in adherent conditions for 24 h in the presence or absence of 0.1 ng/ml LMB1. The percentage of tumourspheres is shown relative to the number of cells seeded. (*p < 0.05, paired t-test, error bars = SEM; all graphs represent means of at least 3 independent experiments)

To investigate the functional consequences of cytoplasmic redistribution of c-FLIP on TRAIL- sensitivity, sub-cellular localisation mutants of cFLIP were generated according to Katayama et al. 2010 [24]. By mutating the nuclear localisation and export sequences of cFLIP, it was possible to generate cFLIP which was preferentially over-expressed in the cytoplasm and nucleus respectively (Fig. 3a and b). Over-expression of cytoplasmic cFLIP was able to protect MCF-7 tumoursphere-forming cells from TRAIL, whereas over-expression of nuclear cFLIP was not protective (Fig. 3c). Furthermore overexpression of cytoplasmic or nuclear cFLIP increased tumoursphere formation significantly (Fig. 3c), suggesting a role for cFLIP in bCSC maintenance.

To confirm the effect of cytoplasmic depletion of cFLIP on TRAIL sensitivity, the CRM1 nuclear transporter inhibitor Leptomycin B (LMB1) was used to sequester cFLIP in the nucleus [24]. Twenty-four hour treatment with 0.1 ng/ml LMB1 reduced cytoplasmic, and elevated nuclear cFLIP levels in the MCF-7 cell line (Fig. 3d and Additional file 1: Figure S3A). Pre-treatment with LMB1 sensitised MCF-7 cells to TRAIL but this result was not statistically significant (Fig. 3e). However, pre-treatment with LMB1 significantly enhanced the sensitivity of tumoursphere-forming MCF-7 cells to TRAIL (Fig. 3f and Additional file 1: Figure S3B). This was also the case in the TRAIL-resistant tumoursphere-forming cells of the SKBR3 and BT474 lines (Additional file 1: Figure S3C and D).

These data show that only cytoplasmic cFLIP can protect against TRAIL whereas nuclear cFLIP cannot, and suggest that re-localisation of cFLIP from the cytoplasmic compartment to the nucleus can sensitise tumoursphere-forming populations to TRAIL.

### cFLIP promotes the canonical Wnt pathway

cFLIP is well-characterised as an inhibitor of the extrinsic apoptosis pathway, a function which requires its availability in the death inducing signalling complex within the cytoplasm [7], however the nuclear function of cFLIP in breast cancer cells is not known. In lung carcinoma cell lines cFLIP promotes Wnt signalling by two separate mechanisms: firstly by preventing the ubiquitylation and consequent degradation of beta-catenin in the cytoplasm, and also by forming a complex with transcription factors in the nucleus to directly promote Wnt-target gene expression [24, 32, 33].

**Fig. 4:**
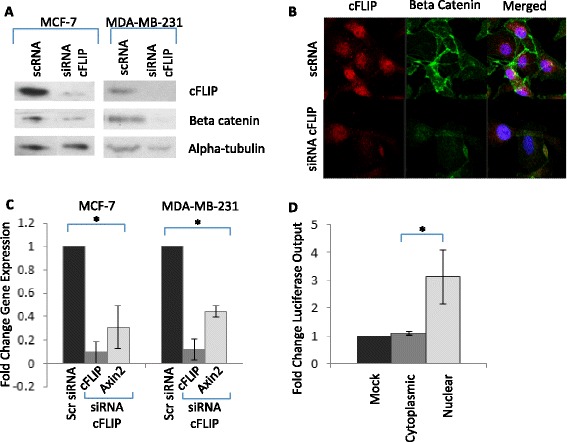
Nuclear cFLIP promotes Wnt-signalling (**a**) Western blotting for cFLIP and beta-catenin performed on cytoplasmic protein extracts of MCF-7 and MDA-MB-231 cell lines transfected with siRNA targeting cFLIP or a scrambled control (loading control alpha-tubulin) (**b**) Immunofluorescence for cFLIP and beta catenin of MDA-MB-231 cells transfected with siRNA targeting cFLIP or a scrambled control (red = cFLIP, green = beta catenin, blue = DAPI) (**c**) qPCR analysis for mRNA levels of cFLIP and Axin2 in MCF-7 and MDA-MB-231 cells transfected with siRNA targeting cFLIP or a scrambled control and cultured in the presence of 10 ng/ml Wnt3a for 48 h. Gene expression is expressed as relative to the untreated control. (**d**) TOPFlash luciferase reporter assay of MCF-7 cells transfected with overexpression constructs; mock (empty), cytoplasmic-localised cFLIP and nuclear-localised cFLIP and cultured in the presence of 10 ng/ml Wnt3a for 48 h. Luciferase output is expressed as relative to that of the mock-transfected cells. (*p < 0.05, paired t-test, error bars = SEM; all graphs represent means of at least 3 independent experiments)

To investigate whether cFLIP was capable of regulating the Wnt pathway in breast cancer cells, the protein levels of beta-catenin were determined following cFLIP inhibition by siRNA in TRAIL-sensitive MDA-MB-231 and TRAIL-resistant MCF-7 cells. Following 48 h transfection, cytoplasmic proteins were analysed for cFLIP and beta-catenin levels by western blotting. Inhibition of cFLIP resulted in a significant decrease in cytoplasmic beta-catenin in both cell lines (Fig. 4a) and this was confirmed by immunofluorescence in MDA-MB-231 cells where a decrease in both membrane-bound and cytoplasmic beta-catenin was observed (Fig. 4b). The cFLIP-mediated regulation of beta-catenin levels also correlated with decreased expression of the Wnt-target gene Axin2 in Wnt3a-stimulated cells [34] (Fig. 4c). Furthermore in Wnt3a-stimulated cells, the Wnt-responsive TOPFlash reporter was activated following over-expression of nuclear but not cytoplasmic cFLIP (Fig. 4d). Taken together these data suggest that cFLIP is a positive regulator of the Wnt pathway and that nuclear but not cytoplasmic cFLIP augments Wnt signalling in MCF-7 and MDA-MB-231 cells.

## Discussion

We have shown that TRAIL is able to suppress the clonal expansion of all six breast cancer cell lines tested, irrespective of their overall sensitivity to TRAIL, and furthermore that TRAIL is able to target directly the stem-like sub-populations in four of these cell lines. Our findings are in accordance with a recent study which showed that bCSCs from basal-like breast cancer cell lines were sensitive to the TRAIL DR5 receptor agonist TRA8 [35]. However this study was limited to only basal-like breast cancer cell lines which are known to be TRAIL-sensitive. We show here that not only the bCSC populations of mesenchymal-like cell lines, but those of the otherwise TRAIL-resistant epithelial-like MDA-MB-468 and MCF-7 lines are also TRAIL-sensitive. These data are important because they widen the range of breast cancer subtypes for which TRAIL can be considered a potential therapeutic and also suggest that conventional assays of cell viability and tumour regression may be insufficient to assess TRAIL efficacy. Liu et al. has shown recently that there exist two stem-like populations; EMT-like and MET-like bCSCs distinguishable on the basis of CD44^+^ and ALDH^+^ expression respectively [22]. We show here that TRAIL targets primarily the CD44^+^, EMT-like subpopulation of bCSCs in the MCF-7 line. We would predict therefore that cell lines such as SKBR3, which is almost 100 % ALDH-positive [36], would be refractory to killing. The data presented in Fig. 1 supports this hypothesis.

Recently it has been reported that MCF-7 cells which had been cultured as 3D tumour spheroids, then disaggregated and seeded in adherent culture were more resistant to TRAIL than their non-spheroid counterparts. Here we have shown that it is the sphere-forming and tumour-initiating MCF-7 cells and their early progenitors specifically that are TRAIL-sensitive. Combined these data suggest that it is the early stem/progenitors themselves, rather than their presence in 3D organoids, that determine TRAIL-sensitivity [37].

We have established one mechanism by which breast cancer cells including bCSCs are rendered TRAIL-sensitive. We have shown that TRAIL sensitivity correlates with reduced cytoplasmic localisation of the endogenous TRAIL pathway inhibitor cFLIP. Cytoplasmic cFLIP levels were particularly low in anoikis-resistant cells which may explain why the combination of cFLIP inhibition and TRAIL treatment results in the complete ablation of bCSCs, and also why it is more effective for bCSCs than the bulk population as siRNA-mediated inhibition of cFLIP would be more efficient in these cases [23].

A number of mechanisms of TRAIL-sensitivity have been described previously in an attempt to explain differences in susceptibility between cancer cells, including differential expression of death receptors DR4 and 5, receptor glycosylation or internalisation, and receptor mutations [38–40]. Interestingly, a recent study has shown that mis-localisation of death receptors can induce TRAIL-sensitivity; however this study revealed that TRAIL-receptor expression or localisation did not correlate with sensitivity across a panel of ten breast cancer cell lines, nor was the TRAIL-susceptibility of stem-like populations addressed. The authors postulated that downstream events instead may be responsible for determination of sensitivity [41]. In terms of regulation of TRAIL-response by cFLIP, its ability to re-localise DISC components away from lipid rafts has been shown to impact on TRAIL-sensitivity of lung carcinoma cell lines [42]. More recently, hyperthermia-induced aggregation and re-distribution of cFLIP from the cytosol to the insoluble cellular fraction further sensitised the MDA-MB-231 line to TRAIL [43]. Whether any of these molecular mechanisms contribute directly to the subcellular redistribution of cFLIP observed in our study remains to be determined.

We also show here that over-expression of nuclear cFLIP, in contrast to cytoplasmic cFLIP is not able to inhibit TRAIL-mediated apoptosis. However we show that nuclear accumulation of cFLIP promotes the Wnt-signalling pathway in breast cancer cell lines. Using the MCF-7 and MDA-MB-231 lines as models of epithelial-like and mesenchymal-like lines respectively we established that inhibition of cFLIP reduced beta-catenin levels and Wnt-target gene expression, whereas over-expression of nuclear cFLIP promoted Wnt-target gene expression. These data are in accordance with previous studies which show that cFLIP promotes Wnt signalling in lung carcinoma cell lines [24, 32, 33] suggesting that cFLIP regulates the Wnt pathway in the same manner in both lung and breast cancer cell lines.

**Fig. 5:**
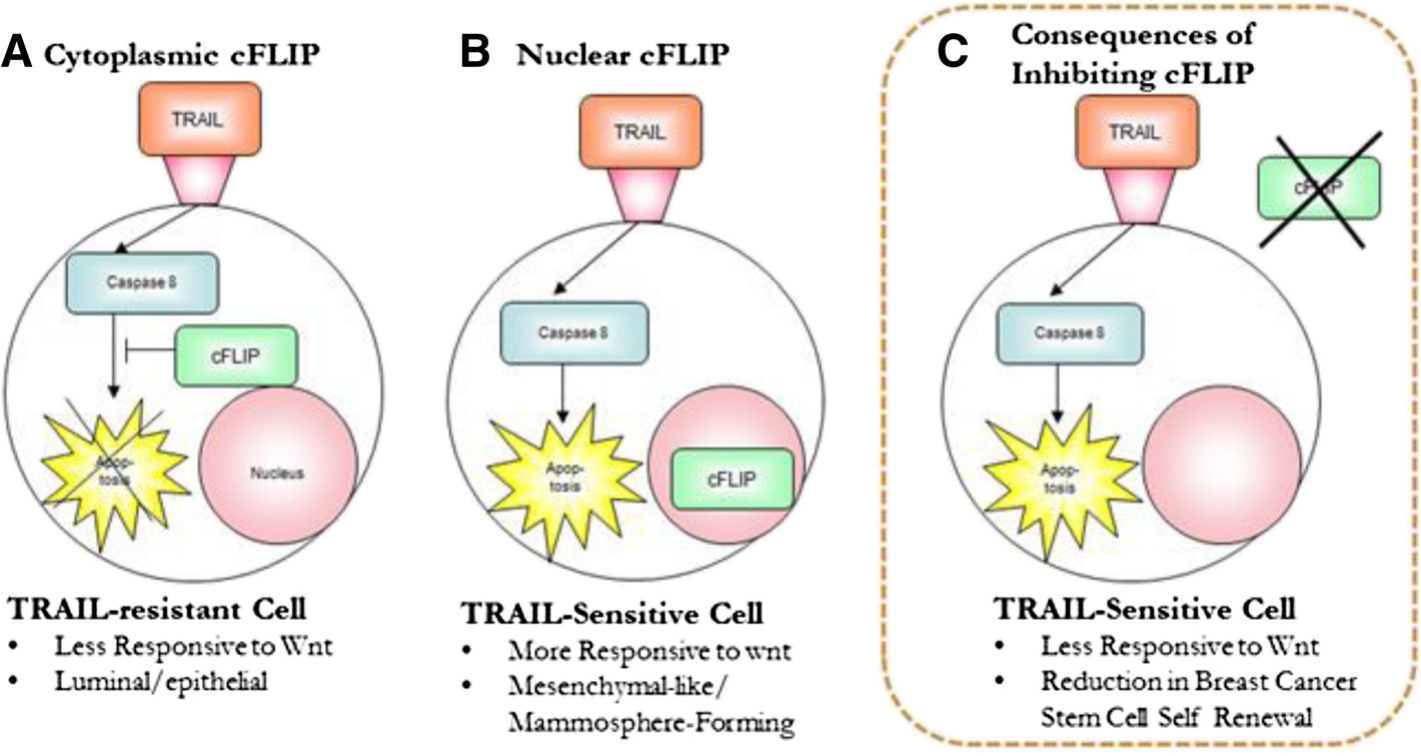
Proposed Model of TRAIL-Susceptiblity in Breast Tumour Subpopulations: (**a**) Epithelial-like cells have cytoplasmic cFLIP and consequently are resistant to TRAIL-mediated apoptosis. (**b**) Mesenchymal-like cells have comparatively reduced cytoplasmic cFLIP and elevated nuclear cFLIP due to its role in the relatively more active Wnt pathway, and are consequently TRAIL sensitive (**c**) The consequences of cFLIP inhibition: sensitisation to TRAIL, reduction in Wnt signalling and potentially a reduction in bCSC self-renewal and proliferation

As TRAIL alone does not completely eradicate tumoursphere-forming or tumour-initiating cells in any breast cancer cell line, we believe our data are evidence of heterogeneity existing in terms of susceptibility to TRAIL. We propose a model to explain the heterogeneous responses of breast cancer cells, including bCSCs, to TRAIL (Fig. 5). In this model, epithelial-like cells (which have reduced levels of Wnt signalling) have abundant cytoplasmic cFLIP and consequently are resistant to TRAIL-mediated apoptosis. In contrast, mesenchymal-like cells have comparatively reduced cytoplasmic cFLIP and elevated nuclear cFLIP associated with its role in the relatively more active Wnt pathway, and are consequently TRAIL-sensitive. Suppression of cFLIP levels therefore has the dual beneficial effects of both sensitizing cells to TRAIL and down-regulating Wnt-signalling (Fig. 5). A down-regulation of Wnt signalling has the added potential to impact negatively on the maintenance of bCSCs [30, 44].

The scope of breast cancer subtypes in vivo which have TRAIL-sensitive bCSCs has yet to be determined. An analysis of cFLIP and TRAIL sensitivity of primary ex vivo tumourspheres in a large panel of tumour samples would help to establish whether such direct correlations exist. Our data suggest that subcellular localisation of cFLIP as opposed to absolute levels may predict response to TRAIL in bCSCs. This means that its efficacy as a biomarker of bCSC response is limited as conventional IHC of pathological sections would not be able to identify the minority bCSC subpopulation. The clinical implication of our findings are that although TRAIL alone is a potential therapy which requires unconventional methods to determine tumour efficacy, a much more effective therapeutic strategy would be to also inhibit cFLIP (Fig. 5), the consequences of which would not just be a sensitisation to TRAIL but also a reduction in Wnt signalling and therefore potentially a reduction in bCSC self-renewal and proliferation.

## Conclusions

In conclusion we have shown that a safe and available therapeutic agent is able to target the stem-like population of six breast cancer cell lines. Sensitivity to TRAIL was mediated by the cytoplasmic levels of the apoptosis inhibitor cFLIP. Our findings suggest a potential for TRAIL in the treatment of breast cancer patients. Further investigation is required to determine the TRAIL-susceptibility of clinical samples ex vivo.

## Additional file

Additional file 1:
**Supplementary figures.** (PDF 2437 kb)

## Abbreviations

ALDH1: aldehyde dehydrogenase 1
bCSC: breast cancer stem cell
cFLIP: cellular FLICE-like inhibitory protein
CSC: cancer stem cell
DISC: death-inducing signalling complex
DR4: death receptor 4
DR5: death receptor 5
LMB1: leptomycin B 1
TRAIL: TNF-related apoptosis inducing ligand
Wnt: wingless-int
