# Supplementary material for: Cytoplasmic levels of cFLIP determine a broad susceptibility of breast cancer stem/progenitor-like cells to TRAIL
Source: Mol Cancer. 2015 Dec 15;14:209. doi: 10.1186/s12943-015-0478-y (PMC4678708; doi:10.1186/s12943-015-0478-y)

# Supplementary Figure 1

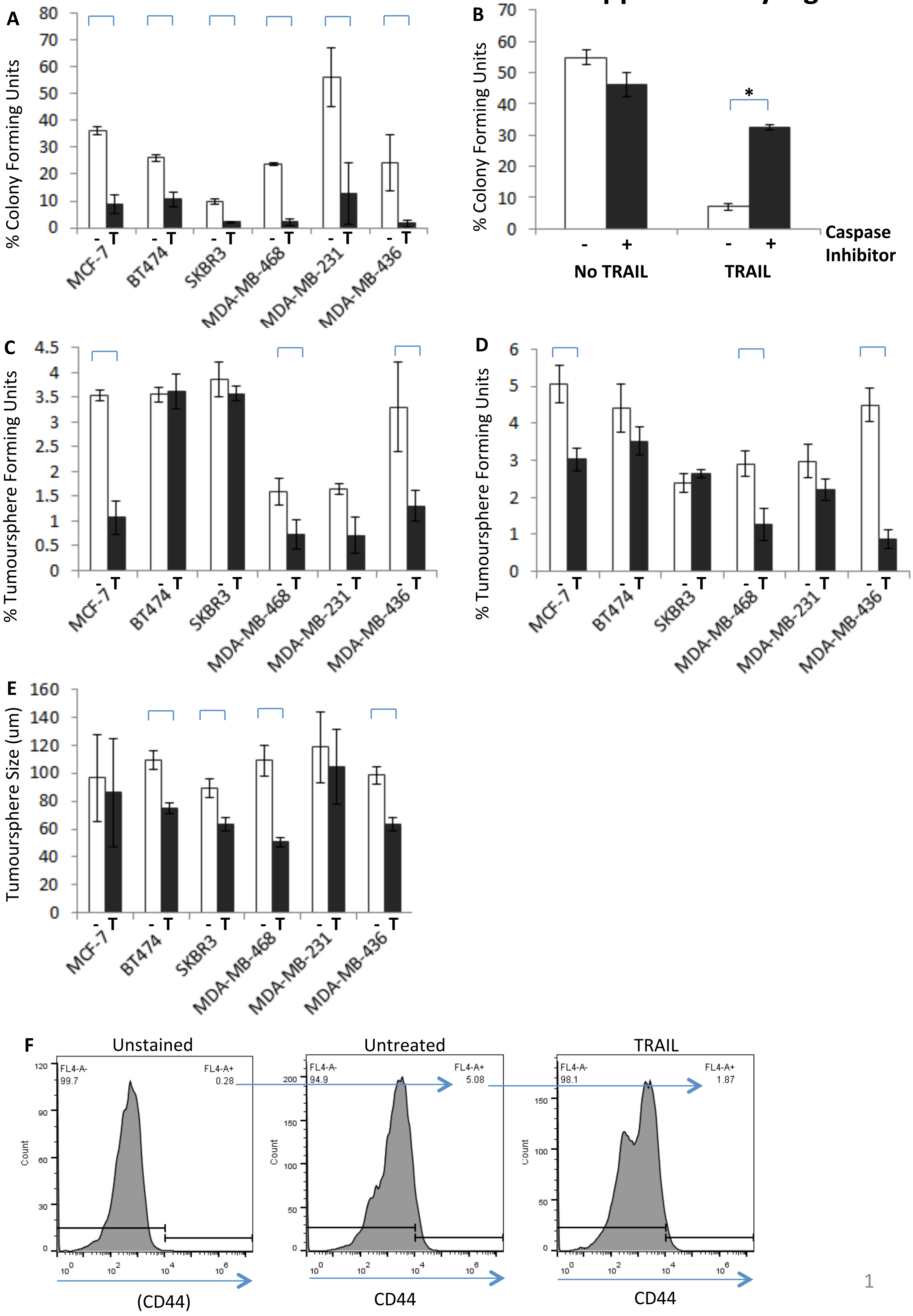

# Supplementary Figure 2

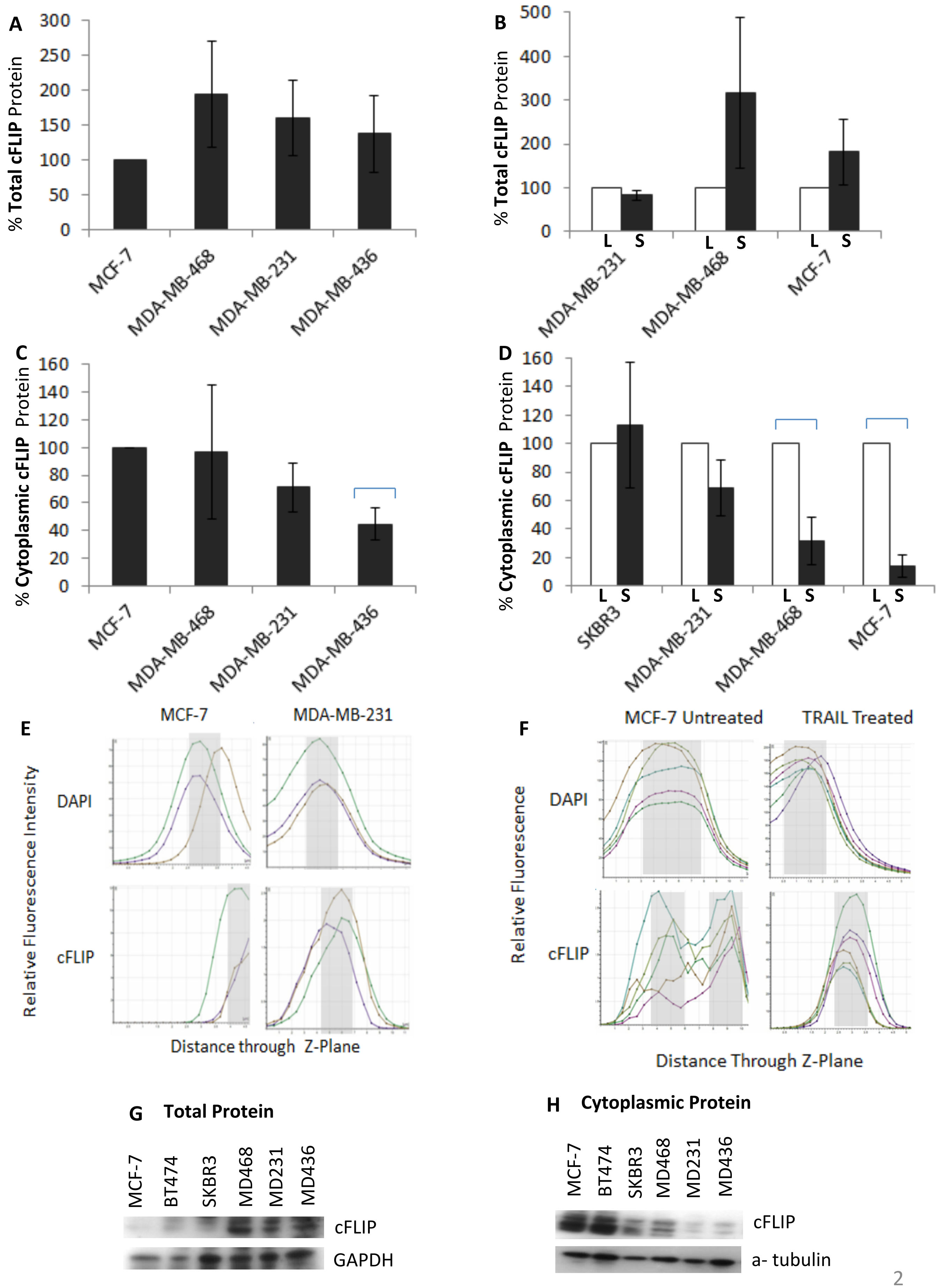

# Supplementary Figure 3

A

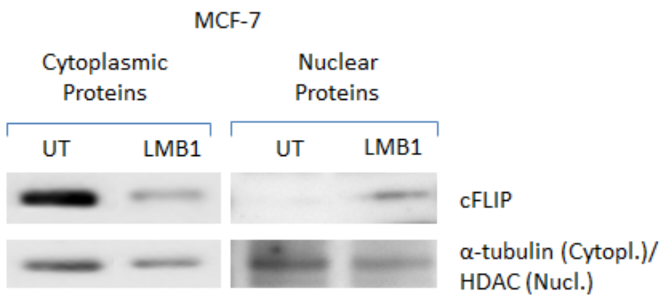


B

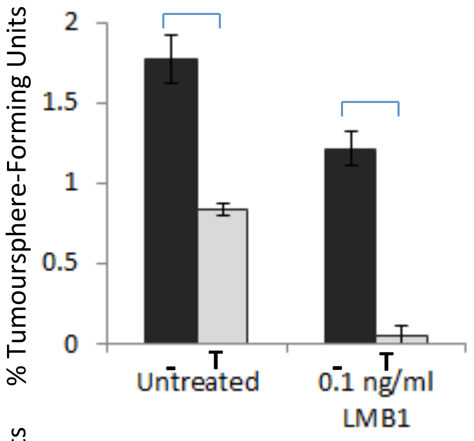


C

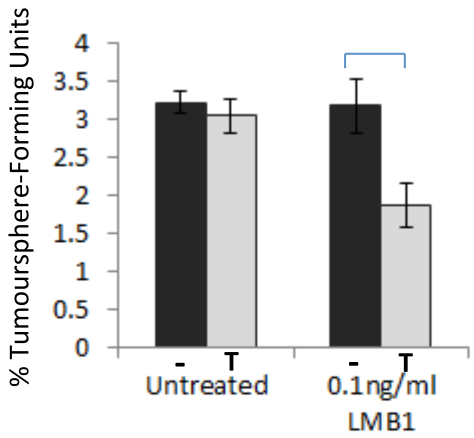


D

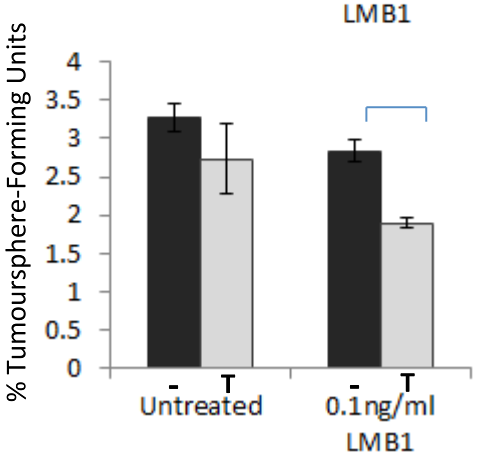

Supplement: Additional file 1: — Supplementary figures. (PDF 2437 kb) [file 12943_2015_478_MOESM1_ESM.pdf]
